# Patients’ and Providers’ Attitudes Toward Artificial Intelligence and Electronic Health Record Use in Deep Phenotyping and Rare-Disease Screening: An Empty Systematic Review

**DOI:** 10.3390/healthcare14142153

**Published:** 2026-07-16

**Authors:** Sylvia Martin, Åsa Grauman, Joshua Coulter, Besir Hasan, Jorien Veldwijk, Mats Hansson, Alain Anyouzoa, Elisabeth Nyoungui, Kaisa Elomaa, Jana Zschuentzsch

**Affiliations:** 1 Center for Research and Bioethics, Uppsala University, 753 10 Uppsala, Sweden; 2 Pfizer, Inc., 66 Hudson Boulevard, New York, NY 10001, USA; 3 Department of Neurology, University Medical Center, 37075 Göttingen, Germanyj.zschuentzsch@med.uni-goettingen.de (J.Z.); 4 Erasmus School of Health Policy & Management, Erasmus University Rotterdam, 3000 DR Rotterdam, The Netherlands; 5 Takeda Oy, 00240 Helsinki, Finland; 6 Institute of Medical Informatics, University Medical Center, 37075 Göttingen, Germany

**Keywords:** electronic health records, rare diseases, artificial intelligence, preferences, attitudes

## Abstract

**Background:** The integration of Artificial Intelligence (AI) and algorithms into healthcare is transformative, particularly in diagnosing rare diseases (RDs), enhancing the accuracy and speed of condition identification. **Objectives:** This systematic literature review investigates perceptions and attitudes toward the use of AI in Electronic Health Records (EHRs) for screening patients at risk of RD, aiming to understand patients’ and healthcare providers’ expectations and concerns. **Methods:** Following PRISMA guidelines, a systematic search was performed in December 2023. A search strategy developed by the research team in collaboration with an expert librarian, using the PICO framework, was applied. Searches were conducted in PubMed, Scopus, and Web of Science. The search strategy covered four main concepts: diagnostic techniques, medical records, AI, and attitudes toward these technologies. **Results:** The initial search retrieved 3348 articles after duplicate removal. However, no studies met the inclusion criteria. As a result, no eligible studies were identified, preventing risk-of-bias assessment or data synthesis. **Discussion:** The absence of relevant studies highlights the need for further research focusing on patient and healthcare provider attitudes toward AI-integrated EHRs, especially in RD and their early detection. **Conclusions:** The lack of studies on stakeholder attitudes toward AI in EHRs for RD screening represents an important research gap. Addressing this gap will improve the understanding and development of AI applications in healthcare, ensuring they meet user needs and ethical standards.

## 1. Introduction

Perspectives from all stakeholders, including patients, healthcare providers, caregivers, and governments, have become increasingly important in medical research since the advent of the PREFER recommendations [1]. For example, in 2021, the World Health Organization (WHO) emphasized the need to integrate patient attitudes into medical development; rare diseases (RDs) are no exception [2,3]. It is important that research meaningfully involves users and providers to support the implementation of findings in clinical settings with the highest probability of success, especially in RD [4]. In recent years, the integration of Artificial Intelligence (AI) into the healthcare sector has represented a transformative shift, particularly in the screening and diagnosis of RDs. These technological advancements offer unprecedented opportunities to enhance the accuracy, efficiency, and speed of diagnosing complex conditions that are often difficult to identify [5]. Rare diseases, characterized by their low prevalence among the population (fewer than 1 in 2000 people) [6], present significant challenges for traditional healthcare methodologies due to their wide variety and overlap with symptoms of common diseases, as well as the heterogeneity of symptom presentation.

AI systems, through machine learning and deep learning algorithms (e.g., natural language processing), can analyze vast datasets far beyond the capacity of human practitioners, identifying patterns and correlations that may elude even the most experienced professionals (see definitions in [7]). This capability is crucial for RDs, where the limited number of cases can hinder the accumulation of comprehensive knowledge through experience alone. By leveraging data from electronic health records (EHRs), genetic information, and even wearable health devices, the use of algorithms in screening processes can streamline the identification of RDs, reducing the time and resources typically required [8]. Reducing time to diagnosis is particularly important given the diagnostic journey many patients with RDs face; many undergo years of misdiagnoses and ineffective treatments. By enhancing diagnostic accuracy and efficiency, AI/ML not only improves patient outcomes but also contributes to a better understanding of RDs, which may facilitate research into new treatments and interventions [9].

International research efforts are focusing on improving screening for RDs in newborns and advancing sequencing technologies. Large initiatives such as the Baby Seq project [10] and the Screen4Care (S4C) project aim to expedite RD diagnosis using genetic testing and digital technologies, including AI. The S4C project (the funding source for this research), for example, is funded by the European Union via an Innovative Medicines Initiative (IMI) project, a global initiative accelerating access to innovative medicines. S4C employs two key strategies: genetic newborn screening (gNBS), targeting selected RDs with treatable or actionable conditions, and innovative digital solutions designed to foster connectivity within RD communities and promote stakeholder involvement for expedited diagnosis [11].

However, the implementation of these technologies is not without challenges. Issues such as data privacy, the need for large and diverse datasets aligned with the FAIR (Findable, Accessible, Interoperable, Reusable) principles to train AI models [12,13], and the integration of AI tools into clinical practice must be addressed [14]. From the perspective of different stakeholders, AI and ML innovations are increasingly viewed as more reasonable, feasible [15], and acceptable [16].

In recent years, preference studies have been developed in the medical and health sectors to assess the impact of users’ and healthcare providers’ experiences. In RDs, the plausibility of AI and ML as aids for detection is increasingly a reality [8,17]. However, ethical issues are more pressing in RDs, as little information can create challenges because of data fragmentation, data quality, and data privacy (e.g., the potential identification of patients due to the “rare” aspect of their diagnosis) [18]. In an area of such high stakes and innovative growth, patient engagement and the inclusion of stakeholder attitudes are warranted and necessary.

To better contextualize the gaps identified in the review, it is important to note that several adjacent research domains are already well developed (particularly over the last 5 years) but remain largely disconnected. Systematic reviews have documented substantial progress in AI-based rare-disease detection and phenotyping using EHR data, particularly through machine learning and natural language processing approaches [19,20]. In parallel, a mature body of literature exists on EHR-driven phenotyping methods, focusing primarily on technical model development and evaluation [21,22]. Separately, stakeholder attitudes toward health data sharing have been extensively synthesized, highlighting concerns about privacy, trust, and secondary data use [23,24]. Finally, a growing number of systematic reviews address acceptance and trust in clinical AI tools, emphasizing the importance of user perspectives for successful implementation [25,26,27].

This literature review aims to investigate the perceptions and attitudes of stakeholders (including healthcare professionals, patients, and the general public) toward the use of AI/ML in electronic health records (EHRs) for screening RDs.

## 2. Methods

This review was performed in accordance with the PRISMA (Preferred Reporting Items for Systematic Reviews and Meta-Analyses) guidelines. The review protocol was registered in the Open Science Framework (OSF) database (Registry number: 10.17605/OSF.IO/SDXP9).

### 2.1. Search Strategy

Together with a university librarian from Uppsala University, three researchers with diverse backgrounds (public health, clinician medicine, and psychology) developed the search strategy in December 2023. Databases were selected to cover both social sciences and medical literature (PubMed, Scopus, and Web of Science). Search terms were developed to encompass the four main concepts: diagnosis and diagnostic techniques, electronic health records, artificial intelligence, and attitudes toward these technologies. The initial search strategy included rare-disease-specific terms with no positive results on the selection criteria. Out of the 260 articles retrieved, none were eligible after title and abstract screening. We hypothesized that the rare-disease terms were too restrictive to capture the subtility of certain rare-disease names and genetic conditions (see Table A1). Therefore, we extended the search strategy while ensuring that all articles retrieved by the initial search were also included in the revised strategy, demonstrating that the expanded search was coherent and did not deviate from the RDs’ associated terms. The final search combined MeSH terms and keywords in titles and abstracts to identify relevant literature.

For the diagnosis and diagnostic techniques domain, the search included terms such as “Diagnosis”[MeSH] OR “diagnosis code*”, “phenotyp*”. The medical records domain included terms such as “medical records”[MeSH Terms] OR “Registries”[MeSH Terms]. The role of Artificial Intelligence was explored using “Artificial Intelligence”[MeSH], supplemented with keywords such as “algorithm*”, “artificial intelligence” in titles and abstracts. Attitudes toward these technologies were investigated using “Attitude”[Mesh] OR “Patient Preference”[MeSH] (see the full search strategy in Table A2). The search strategy employed logical operators to combine these concepts, ensuring that articles retrieved were relevant across all domains.

**Figure 1:**
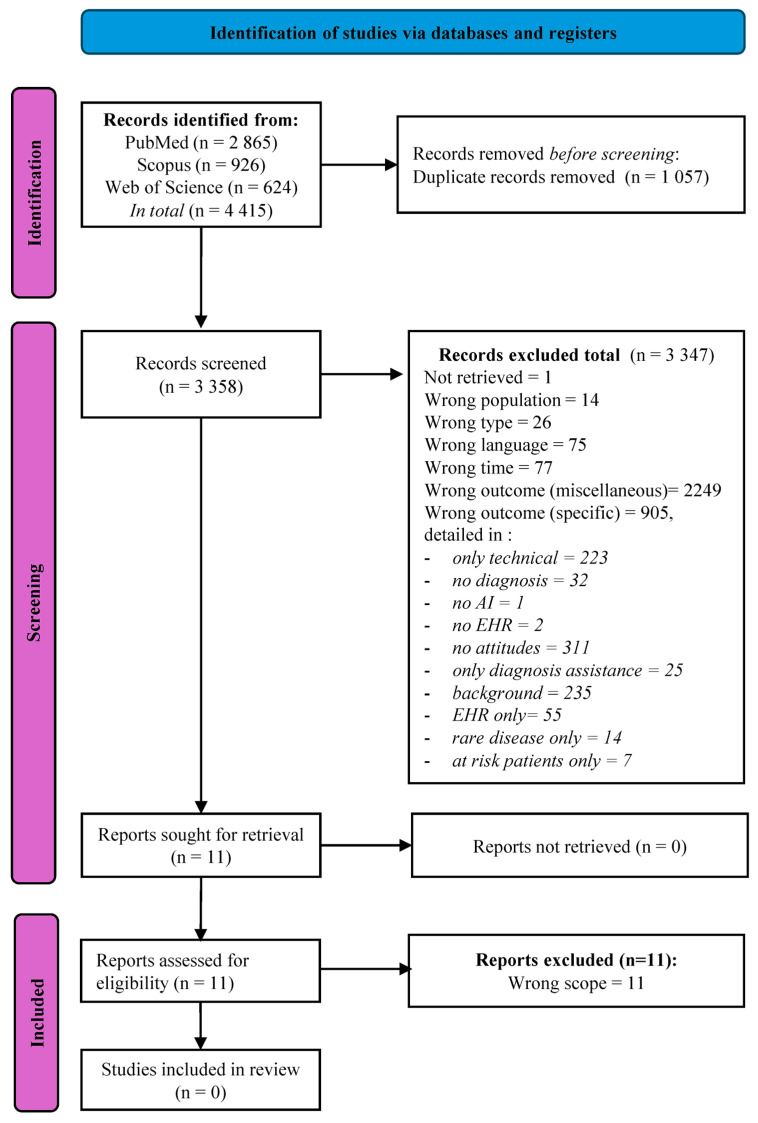
Prisma flowchart.

Professional librarians independently reviewed the search strategy to ensure consistency of key words and MeSH terms across the different databases. This approach was designed to capture a comprehensive set of articles at the intersection of AI, healthcare data management, and the human factors influencing the adoption and effective implementation of these technologies in healthcare settings (see search strategy in Table A2). The methods used in this review followed current guidelines and recommendations [28] (see Figure 1 for the PRISMA Flowchart). In addition, the reporting structure was informed by previously published systematic reviews with null findings [29].

### 2.2. Eligibility Criteria

To be included in the study, studies must have met all the following criteria:

- Full text was available.
- Written in English.
- Reported empirical research (quantitative or qualitative or mixed methods).
- Included stakeholders of interest (patients, the general public, or healthcare professionals).
- Addressed all four of the following concepts:
  ○ (1) Deep learning or algorithms;
  ○ (2) Detection of diagnosis, diseases, or at-risk alerts for RD;
  ○ (3) Use of Electronic Health Records;
  ○ (4) Consideration of attitudes or preferences of users or healthcare providers.

All RDs were considered according to the European definition adopted by Orphanet, which states “In Europe, a disease is considered to be rare when it affects 1 (or less) person per 2000” [6]. The review included both early-onset RDs and ultra-RDs. This broad approach was intended to capture a wide range of research for inclusion, including all RDs that may be suitable for screening using digital health technologies. Studies without abstracts were included during the initial screening stage.

The exclusion criteria were:

- Conference and study protocols;
- Theoretical papers;
- Editorials;
- Research on animals;
- Studies focused on medical procedures;
- Technical development of AI/ML;
- Performance evaluations of diagnostic tools;
- Studies focused on treatment decisions or medication changes.

**Figure 2:**
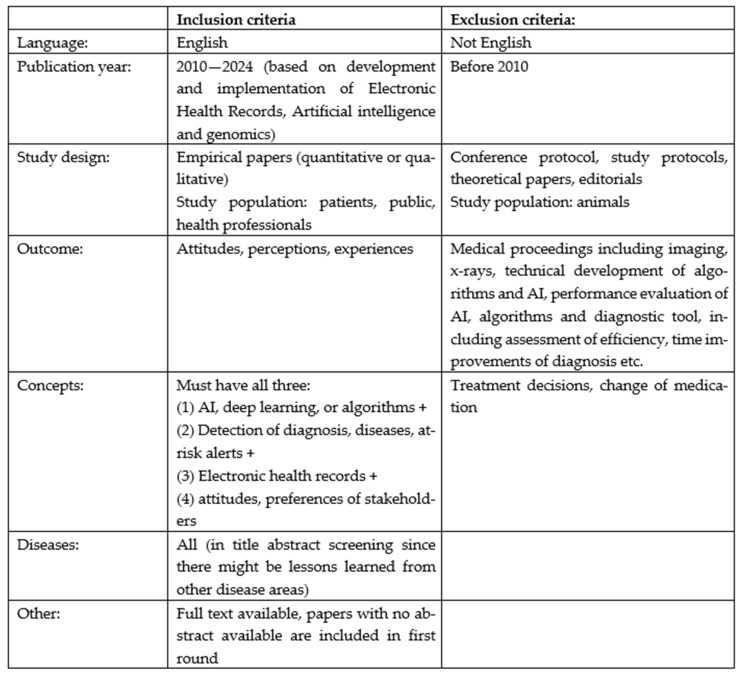
Inclusion and exclusion criteria applied during title/abstract and full-text screening.

This approach ensured a thorough review of the literature relevant to technology’s role in healthcare, emphasizing the significant impact of Electronic Health Records and AI on healthcare delivery and perceptions (see Figure 2, Inclusion–Exclusion table). We therefore restricted the inclusion timeline to articles published from 2010 to 2024 for double blind review. However, articles published before 2010 were also screened by a single reviewer to ensure that no relevant paper was misclassified.

### 2.3. Selection Process

After the initial search, 4415 records were identified. Following the removal of duplicates, 3358 articles remained for screening. Using the rayyan.ai software (free access version without AI companion), all articles were independently screened in double-blind manner based on their titles and abstracts by four reviewers (SM, ÅG, BA, JC). Title and abstract screening adhered to the predefined inclusion and exclusion criteria, aimed at identifying studies assessing participants’ attitudes or preferences regarding the use of Electronic Health Records and Artificial Intelligence. Final decisions on inclusion and exclusion were reached through consensus among at least three reviewers, with final confirmation by the principal investigator to ensure that all selection was harmonized across reviewers’ competences and experiences. Because one reviewer was a medical doctor, articles with technical medical vocabulary were referred to that reviewer to support the final decision.

After title and abstract screening, 11 articles remained and underwent full-text review. Full-text screening was also conducted independently by two reviewers using the same consensus process. Ultimately, none of the studies were included in the results because they did not meet the inclusion criteria (see Table 1 and Figure 1 for detailed reasons for exclusion). Therefore, even though scientific quality appraisal (scoring) was planned using Joanna Briggs Institute (JBI) evidence-based healthcare tools, this part of the analysis was not performed.

### 2.4. Outcomes

The primary outcome of interest was stakeholder attitudes and preferences regarding the use of Artificial Intelligence (AI) and Electronic Health Records (EHRs) in the context of rare-disease screening.

Outcomes of interest included, but were not limited to:

- Perceived acceptability of these screening technologies;
- Trust in AI systems;
- Perceived usefulness of these technologies;
- Willingness to use or adopt such technologies;
- Perceived risks and ethical concerns.

## 3. Results

No studies met all the predefined inclusion criteria. Several articles were noted as related to (1) deep learning or algorithms; (2) detection of diagnosis, diseases, or at-risk alerts for RD; (3) the use of Electronic Health Records; and (4) consideration of attitudes or preferences of users or healthcare providers (see Table 1). Even in the shortlisted articles that underwent full-text review, most of them were excluded due to the absence of (2) detection of diagnosis, diseases, or at-risk alerts for RD. The second most frequent reason was the lack of (3) Electronic Health Records, and only 1 was excluded because it did not (4) consider attitudes or preferences of users or healthcare providers. All search results are publicly available through the OSF at https://doi.org/10.17605/OSF.IO/ZYMTW.

## 4. Discussion

Our findings highlight a significant gap in the literature and the limited exploration of preferences in a prolific research area. Specifically, more research is needed at the intersection of (1) deep learning or algorithms; (2) detection of diagnosis, diseases, or at-risk alerts for RD; (3) the use of Electronic Health Records; and (4) consideration of attitudes or preferences of users or healthcare providers. Given the rapid growth of technical and clinical research on the use of AI and ML with EHR data for RD screening, evidence on stakeholder perspectives is urgently needed.

Qian et al. (2021) explored the landscape and “hot topics” in the EHR literature and pointed at the phenomenal growth in publications since 2004, allowing them to analyze more than 10,000 articles [41]. They analyzed the main content topic clusters and identified key terms in the main nodes that were used in the development of this study. The authors grouped machine learning and AI under the broader heading of “technology.” Similarly, they classified genetics under “subjects.” Concepts related to attitudes and perceptions were classified as “information system researches.” Finally, patient satisfaction was categorized under “patient engagement.” This specific clustering of patient-centered and technical concepts may be a key factor in understanding the study’s findings.

References to patient preferences in the existing literature mainly focus on the technical aspect of experience or on the ethical debate surrounding AI/ML and EHR data. Consequently, the more complex topic of gNBS for RDs has not yet been explored. In terms of research subjects, “children” and “adolescents” were considered but newborns were not included. Acceptance and stakeholder preference for EHR use in the context of RDs has been examined primarily from the perspectives of data sharing and protection [42,43,44]; however, the use of AI/ML has not yet been thoroughly investigated.

If we relate our findings to the literature presented in the background, the existing evidence clearly maps the gap identified in this review. Of the systemic reviews published over the last five years, none has addressed the intersection of these domains considered in our review (see Table 2).

### 4.1. Identified Challenges

The lack of evidence on stakeholder perspectives and preferences regarding the use of EHR with AI or ML for identifying undiagnosed RDs may reflect the inherent complexity of both RD research and its ethical considerations. Research on RDs is challenging due to the small number of eligible participants [45] and, on a technical level, the need for large volumes of interoperable data to avoid bias in AI or ML models [46,47]. Moreover, there is an important distinction between suggesting a patient is at risk for a diagnosis and ruling out a diagnosis. This distinction may have influenced our search results, as most research do not differentiate between the terms “screening”, “testing”, or “diagnosing.” Most research focus on diagnosing symptomatic patients, whereas “flagging” or screening is a question of detecting potential risk for a disease, sometimes in the absence of classical symptoms—especially in newborns. This can be further complicated by the fact that EHR data are not specifically designed for use beyond the NBS period [48]. Similar issues exist for the terminology and usage of the key concept “AI.” The evolution and conceptualization of AI literacy is still an issue that has direct consequences for both research and society [49]. For example, AI is increasingly integrated into user-facing technologies; however, users are often told that the software they interact with relies on an “algorithm” that recognizes preferences and behavior. Such *“misconceptions can limit people’s ability to effectively use, collaborate with, and act as critical consumers of AI”* ([49], p1). In 2019, Wang attempted to clarify the definition of artificial intelligence beyond the classical “intelligent machine”, expanding it to encompass cognitive tasks, notably learning and problem-solving, leveraging cutting-edge technological advancements such as machine learning, natural language processing, and neural networks [50].

### 4.2. The Importance of “Empty Reviews”

Our results also provide an opportunity to contribute to the methodological discussion on the value of publishing null results or “empty reviews.” In the field of pharmacopsychology, it is well recognized that the non-publication of null results can distort the evidence base and introduce bias into subsequent research [51], adding to the broader “catalogue of biases” in scientific publishing [52]. At the methodological level, there is ongoing debate regarding the usefulness of “empty reviews” [53], with arguments often focusing on their prevalence within a specific field. In the fields of AI and RD, such publications are not commonly reported. On a research ethics level, while preventing publication bias is important, it is also vital to consider the need to avoid duplication of efforts [54], enhance methodological transparency [55], and thus, promote research integrity and informed decision-making [56]. Moreover, sharing null results can save other researchers time and resources, particularly in contexts where research findings are not consistently published across studies [57]. In an open data system, researchers still need to triage datasets and methods that are unlikely to yield meaningful results [58]. Highlighting the lack of research in a certain field (i.e., identifying large research gaps) can help researchers focus on areas of interest more rapidly [59]. In this context, the present study makes a methodological contribution by formalizing the use of an “empty review” in a rapidly evolving field. Such reviews can help identify neglected areas, guide research priorities, and improve transparency while reducing publication bias and duplication of effort [60,61]. This is particularly valuable in fast-moving domains such as AI in healthcare, where high publication volume can obscure important gaps in the evidence base. By clearly demonstrating a missing intersection of evidence, this review frames the absence of studies as a meaningful and actionable finding.

Moreover, the study highlights a key methodological issue: inconsistent terminology. The conflation of terms such as “screening”, “diagnosis”, and “risk detection” can obscure relevant evidence and hinder systematic retrieval. As recognized in healthcare research, such variability limits comparability and synthesis across domains [62]. Our findings suggest that part of the observed gap may stem from terminology-driven invisibility rather than a true absence of related work.

### 4.3. Novelty and Contribution

Existing literature has examined AI implementation, stakeholder perspectives, and EHR-based clinical applications largely in isolation, with many studies adopting a predominantly technical focus [63,64]. However, qualitative syntheses of clinical AI highlight the importance of stakeholder engagement while also identifying persistent gaps in understanding how these perspectives influence implementation outcomes [25]. Similarly, broader research on AI adoption consistently identifies trust, organizational readiness, and ethical concerns as central barriers in real-world stakeholder interaction—factors that are essential for effective implementation [64,65,66]. Yet these considerations are rarely examined in relation to specific use cases such as RD screening [67,68]. This imbalance is reflected in the broader AI literature, where evaluations tend to prioritize accuracy and efficiency over experiential, ethical, and social dimensions, and where patient perspectives remain marginal [69]. By explicitly combining AI/ML, EHR use, RD detection, and stakeholder preferences within a single framework, this review introduces a multi-dimensional gap-mapping approach that has not been systematically applied in prior work.

A second key contribution is the identification of a translational disconnect between technological development and real-world implementation. Although AI innovation is advancing rapidly, relatively few systems are successfully integrated into routine clinical practice [25,68,70]. In the RD context, AI has been framed as transformative across the patient journey; however, this vision presumes alignment with stakeholder expectations that remains underexplored [71]. This review provides structured evidence that such alignment is currently lacking, extending the concept of the research-to-practice gap to include stakeholder-informed AI deployment.

### 4.4. Limitations

The findings of this systematic review should be interpreted in light of several methodological considerations that may partly explain the absence of eligible studies. While our results suggest a lack of evidence at the intersection of AI/ML, EHR use, RD detection, and stakeholder preferences, this may also reflect potential limitations in evidence retrieval. Terminology heterogeneity could have played a significant role, as concepts in this field—such as “screening”, “early detection”, “phenotyping”, “flagging”, and “diagnosis”—are often used interchangeably across disciplines. This variability may have reduced the sensitivity of the search strategy across databases. Similarly, variability in how “artificial intelligence” is described (e.g., algorithms, machine learning, decision support systems) may have contributed to incomplete retrieval, despite efforts to use broad search terms. Moreover, the review was restricted to English-language publications, which may have introduced language bias [72,73] particularly given the global nature of rare-disease research and AI development. Although major databases were included, relevant studies indexed in specialized technical repositories (e.g., computer science databases) or other interdisciplinary sources may not have been captured. Finally, the exclusion of grey literature—often omitted in SLR in the medical field—may have limited the scope of identified evidence, particularly in the field of AI, where innovations are often first reported outside traditional academic publishing channels.

### 4.5. Future Research Directions

This review highlights an urgent need for empirical, stakeholder-centered research at the intersection of AI/ML, EHR-based screening, and rare diseases (RDs). Future work should move beyond technical performance to examine the perspectives of patients, caregivers, and healthcare providers, particularly in pre-diagnostic contexts marked by high uncertainty—areas still underrepresented in current research and governance frameworks [72,74,75]. Priority areas include: (1) acceptance thresholds for risk-flagging versus formal diagnosis, (2) preferences regarding secondary use of EHR data, consent models, and data governance, and (3) the perceived psychosocial impact of being identified as “at risk” for an RD. Given that willingness to share health data varies by context and is shaped by trust, transparency, and perceived benefit [23], further research should also examine attitudes toward data reuse, linkage, and cross-institutional sharing, alongside concerns about privacy, representativeness, and data quality [76]. Mixed-methods and participatory study designs will be essential, with explicit comparisons across stakeholder groups.

In parallel, future research should explicitly integrate principles of explainable AI (XAI) into preference studies. The “black-box” nature of many AI systems remains a major barrier to trust and adoption, and explainability has been identified as critical for improving interpretability, accountability, and clinical usability [77,78]. Empirical studies should assess how transparency and communication of uncertainty influence trust, particularly in EHR-based systems that rely on complex and imperfect data. Testing different explanation formats (e.g., feature attribution methods, narratives, and probabilistic outputs) and varying levels of detail will be crucial to determine what is meaningful and acceptable to diverse users [74,79]. Embedding XAI within user-centered design approaches is essential to ensure that EHR-based AI tools are not only accurate but also interpretable, trustworthy, and aligned with stakeholder expectations.

## 5. Conclusions

In conclusion, this study highlights a significant gap in the literature regarding stakeholder preference studies in the context of RD screening and, consecutively, the use of EHR data for RD detection). This gap stands in contrast to the extensive body of research on technical and clinical aspects of AI and ML applications. The limited focus on stakeholder preferences in EHR-based AI use underscores the methodological complexity and ethical challenges inherent in this area. This gap is further compounded by the small number of eligible participants in RD research and the inconsistencies in terminology, particularly the overlap between screening and diagnosis. Additionally, the publication of null results, as demonstrated by our findings, is crucial for enhancing methodological transparency, reducing publication bias, and promoting research integrity. Addressing these gaps and challenges is essential to advance the field and support informed decision-making in the development and implementation of emerging healthcare technologies.

## Figures and Tables

**Table 1:** Selected articles for full-text assessment and final reasons for rejection.

Authors	Year	Title	Resume	Reason for Being Shortlisted Before Exclusion	Inclusion Criteria Being Presented: (1) Deep Learning or Algorithms; (2) Detection of Diagnosis, Diseases, or At-Risk Alerts for RD; (3) The Use of Electronic Health Records; (4) Consideration of Attitudes or Preferences of Users or Healthcare Providers	Exclusion Reason
1. Albrink et al. [30]	2024	Usability of an app for medical history taking in general practice from the patients’ perspective: cross-sectional study	Assessing the usability of a medical history app from the patient’s perspective in general practice settings. They recruited 406 patients with acute complaints across eight clinics. Participants used the app during their waiting time and then completed the System Usability Scale (SUS), a standardized 10-question survey. The analysis used both descriptive and inferential statistics to identify patient characteristics that correlated with higher or lower SUS scores.	Article about direct feedback from patients regarding the implementation of new technologies, interest in the system usability scale measurement used in the research	(1) deep learning or algorithms (3) the use of Electronic Health Records (4) consideration of attitudes or preferences of users or healthcare providers	Absence of (2) detection of diagnosis, diseases, or at-risk alerts for RD
2. Funes Hernandez et al. [31]	2024	Design and Implementation of an Electronic Health Record-Integrated Hypertension Management Application	Hypertension management platform for HCPs. Several key stages: needs assessment, clinical workflow analysis, creation of treatment algorithms, platform design, and integration with EHR. To identify specific needs and analyze clinician workflow. Interviewed and surveyed five Stanford clinicians from primary care and cardiology, along with their clinical care teams (nurses, advanced practice providers, and medical assistants).	Article about a vast variety of HCPs regarding use of digital technologies, key stage approaches informing the process.	(1) deep learning or algorithms (4) consideration of attitudes or preferences of users or healthcare providers	Absence of (2) detection of diagnosis, diseases, or at-risk alerts for RD and (3) the use of Electronic Health Records
3. Jauk et al. [32]	2021	Technology Acceptance of a Machine Learning Algorithm Predicting Delirium in a Clinical Setting: a Mixed-Methods Study	Evaluate HCPs’ acceptance of an existing ML application designed to predict delirium risk in inpatients. Mixed-methods approach to gather feedback from physicians and nurses (47 participants) who regularly used the app. The evaluation was framed by the Technology Acceptance Model, focusing on perceived ease of use, perceived usefulness, actual system use, and output quality.	Article about HCP acceptance of machine learning tools, notable for use of the Technology Acceptance Model on a specific field (narrowed population of delirium affected patients).	(1) deep learning or algorithms (4) consideration of attitudes or preferences of users or healthcare providers	Absence of (2) detection of diagnosis, diseases, or at-risk alerts for RD and (3) the use of Electronic Health Records
4. Jones et al. [33]	2019	Public Views on Models for Accessing Genomic and Health Data for Research: Mixed Methods Study	Social acceptability of different access models for reusing genomic data collected for research in combination with health data. Eight public workshops (n = 116 participants) were performed. Participants preferred consent-based access and data use within a safe haven over external release or open access. Specific perceived risks included the potential for data use by unscrupulous parties with open access, data security concerns with external release, and the need for robust safeguards.	Public views on models accessing genomic data and expression of concern in a large sample in the original format of the workshops.	(1) deep learning or algorithms (3) the use of Electronic Health Records (4) consideration of attitudes or preferences of users or healthcare providers	Absence of (2) detection of diagnosis, diseases, or at-risk alerts for RD
5. Lærum et al. [34]	2014	A taste of individualized medicine: physicians’ reactions to automated genetic interpretations	Physicians’ reactions to automated interpretations of genetic tests within EHR. These qualitative findings were then triangulated with responses from a survey. While the algorithm’s explanation was prominently placed in the user interface for transparency, this design choice led to considerable confusion. Findings suggest that background information and references should be available but less prominent than the actual results and recommendations.	HCPs’ reactions to automated genetic interpretation, narrowing the topic to genetics and originality of the patient’s scenario-based methods	(1) deep learning or algorithms (3) the use of Electronic Health Records (4) consideration of attitudes or preferences of users or healthcare providers	Absence of (2) detection of diagnosis, diseases or at-risk alerts for RD
6. Mo et al. [35]	2015	Desiderata for computable representations of electronic health records-driven phenotype algorithms	Clinicians and informaticians developed ten desired characteristics for a flexible, computable Phenotype Representation Model (PheRM) by reviewing common features of multisite phenotype algorithms, existing phenotype representation platforms, and well-known diagnostic criteria and clinical decision-making guidelines.	HCPs need to consider phenotyping tools using EHR for the developed technologies to be clinically relevant but also technically sound	(1) deep learning or algorithms (3) the use of Electronic Health Records (4) consideration of attitudes or preferences of users or healthcare providers	Absence of (2) detection of diagnosis, diseases, or at-risk alerts for RD
7. Pathak et al. [36]	2013	Electronic health records-driven phenotyping: Challenges, recent advances, and perspectives	The Human Genome Project and subsequent genomic research have ushered in an era of individualized medicine. While altered phenotypes are reliable indicators of altered gene functions, EHRs offer a solution to accelerate clinical research and genomic medicine, but they are currently limited by a lack of validated processes and tools for accurate and rapid phenotype extraction. Combination of top-down knowledge engineering and bottom-up learning and analysis is proposed.	Article summarizing the obstacles to phenotyping techniques using EHR and AI in the current context	(1) deep learning or algorithms (3) the use of Electronic Health Records	Absence of (2) detection of diagnosis, diseases, or at-risk alerts for RD and (4) consideration of attitudes or preferences of users or healthcare providers
8. Richesson et al. [37]	2013	Electronic health records based phenotyping in next-generation clinical trials: A perspective from the NIH health care systems collaboratory	The NIH Common Fund’s Health Care Systems Collaboratory program, launched in 2012, comprises seven demonstration projects and seven problem-specific working group “Cores” dedicated to leveraging real-world healthcare data to enhance trial efficiency, relevance, and generalizability. Specific focus on its Phenotype, Data Standards, and Data Quality Core. Presents early observations from researchers conducting clinical trials within large healthcare systems and identifies existing knowledge gaps.	Perspective about the EHR-based phenotypes from the perspective of having “real-world” healthcare data put to use.	(1) deep learning or algorithms (4) consideration of attitudes or preferences of users or healthcare providers	Absence of (2) detection of diagnosis, diseases, or at-risk alerts for RD and (3) the use of Electronic Health Records
9. Sewitch et al. [38]	2012	Qualitative study of physician perspectives on classifying screening and non-screening colonoscopy using administrative health data: adding practice does not make perfect	Qualitative study to identify administrative health database variables that could differentiate between screening and non-screening colonoscopies. Forty family physicians and seven gastroenterologists participated in five specialty-specific focus group sessions. The findings revealed wide variability in clinical and billing practices, which is likely to prevent the development of a highly accurate screening colonoscopy algorithm.	Perspective study on HCPs’ views about screening and non-screening technology using administrative health data	(1) deep learning or algorithms (3) the use of Electronic Health Records (4) consideration of attitudes or preferences of users or healthcare providers	Absence of (2) detection of diagnosis, diseases, or at-risk alerts for RD
10. Smith et al. [39]	2023	“Currently flying blind” Stakeholders’ perceptions of implementing statewide population-based cancer staging at diagnosis into the Western Australian Cancer Registry: a rapid qualitative process evaluation of the WA Cancer Staging Project	Key stakeholders’ perceptions regarding the implementation of cancer staging using Natural Language Processing (NLP) and ML. Data analysis also incorporated Framework Analysis and an adapted visual traffic-light labeling system to grade qualitative data and highlight levels of positivity, negativity, and implementation concern.	Innovative stakeholder-perception analysis in the field of detection of diseases using natural language processing and machine learning.	(1) deep learning or algorithms (4) consideration of attitudes or preferences of users or healthcare providers	Absence of (2) detection of diagnosis, diseases, or at-risk alerts for RD and (3) the use of Electronic Health Records
11. Zhang et al. [40]	2021	Effect of AI Explanations on Human Perceptions of Patient-Facing AI-Powered Healthcare Systems	Evaluate how different types of AI explanations influence people’s perceptions of AI-powered healthcare systems. Large-scale experiment with 3423 participants to assess these effects in the context of comprehending radiology reports. Information about the AI model’s performance can enhance people’s trust and perceived usefulness of the system’s outputs. Conversely, providing local explanations for the rationale behind a prediction can improve understandability but does not necessarily increase trust.	Perception of AI tools in healthcare on a large sample and display of the role of trust and transparency to enhance performances and uptake.	(1) deep learning or algorithms; (4) consideration of attitudes or preferences of users or healthcare providers	(2) detection of diagnosis, diseases, or at-risk alerts for RD; (3) the use of Electronic Health Records; and

Note: Inclusion criteria being presented (1) deep learning or algorithms; (2) detection of diagnosis, diseases, or at-risk alerts for RD; (3) the use of Electronic Health Records; and (4) consideration of attitudes or preferences of users or healthcare providers.

**Table 2:** Comparison of recent systemic reviews and identified research gaps.

Domain	Example Review	Focus	Key Gap
AI for RD detection	Kim 2026 [19]	NLP, ML for diagnosis	No stakeholder perspective
EHR phenotyping	Yang 2023 [21]	ML methods	Technical focus
Data sharing attitudes	Cascini 2024 [23]	Trust, privacy	Not AI-specific
AI acceptance	Hogg 2023 [25]	Implementation, trust	Not RD/EHR combined

## Data Availability

No new data were created or analyzed in this study. Data sharing is not applicable to this article.

## Appendix A

**Table A1:** Restricted search strategy.

Search Number	Search Terms	Number of References
1	“Diagnosis”[Mesh] OR “Diagnostic Techniques and Procedures”[Mesh:NoExp] OR “diagnosis” [Subheading]	10,622,434
2	“classification of disease*”[Title/Abstract] OR “diagnosis code*”[Title/Abstract] OR phecode*[Title/Abstract] OR phenotyp*[Title/Abstract] OR “icd code”[Title/Abstract:~3] OR “icd codes”[Title/Abstract:~3] OR “icd coding”[Title/Abstract:~3] OR “dsm code”[Title/Abstract:~3] OR “dsm codes”[Title/Abstract:~3] OR “dsm coding”[Title/Abstract:~3]	762,132
3	#1 OR #2	11,150,598
4	“Artificial Intelligence”[Mesh]	185,032
5	algorithm*[Title/Abstract] OR “artificial intelligence”[Title/Abstract] OR automat*[Title/Abstract] OR “computational intelligence”[Title/Abstract] OR “data mining”[Title/Abstract] OR “deep learning”[Title/Abstract] OR “deep phenotyp*”[Title/Abstract] OR “digital phenotyp*”[Title/Abstract] OR “inferential statistic*”[Title/Abstract] OR “machine intelligence”[Title/Abstract] OR “machine learning”[Title/Abstract] OR “natural language process*”[Title/Abstract] OR “language prediction model*”[Title/Abstract] OR “large language model*”[Title/Abstract] OR “neural network*”[Title/Abstract] OR “statistical inference*”[Title/Abstract]	802,097
6	#4 OR #5	862,630
7	“Attitude”[Mesh] OR “Patient Preference”[Mesh] OR “Physician-Patient Relations”[Mesh] OR “Clinical Decision-Making”[Mesh]	705,236
8	acceptance OR attitude* OR barrier* OR concerns OR emotion* OR expectation* OR experience* OR facilitat* OR feedback OR feeling* OR interview* OR opinion* OR perception* OR perspective* OR “point of view*” OR preference*	4,202,979
9	#7 OR #8	4,560,493
10	#3 AND #6 AND #9	56,711
11	“Rare Diseases”[Mesh]	14,201
12	“rare disease”[Title/Abstract:~2] OR “rare diseases”[Title/Abstract:~2] OR “rare disorder”[Title/Abstract:~2] OR “rare disorders”[Title/Abstract:~2]	96,212
13	#11 OR #12	104,896
14	#10 AND #13	260

(*) is a truncation (wildcard) symbol. It tells the database to search for all words that begin with the specified root and have any ending.

**Table A2:** Search strategy.

**PubMed** **, 22 January 2024**			
**Search no**	**Search terms**	**Results**	**Comments**
1	“Diagnosis”[Mesh] OR “Diagnostic Techniques and Procedures”[Mesh:NoExp]	9,466,533	
2	“classification of disease*”[Title/Abstract] OR “diagnosis code*”[Title/Abstract] OR phecode*[Title/Abstract] OR phenotyp*[Title/Abstract] OR “icd code”[Title/Abstract:~3] OR “icd codes”[Title/Abstract:~3] OR “icd coding”[Title/Abstract:~3] OR”dsm code”[Title/Abstract:~3] OR “dsm codes”[Title/Abstract:~3] OR “dsm coding”[Title/Abstract:~3]	765,874	
3	#1 OR #2	10,026,051	
4	“Medical Records”[MeSH Terms] OR “Registries”[MeSH Terms]	274,289	
5	“clinical data”[Title/Abstract] OR “clinic notes”[Title/Abstract] OR “clinical notes”[Title/Abstract] OR “progress notes”[Title/Abstract] OR “diagnosis code*”[Title/Abstract] OR “doctors notes”[Title/Abstract] OR “EHR”[Title/Abstract] OR “EMR”[Title/Abstract] OR “health data”[Title/Abstract] OR “health electronic record*”[Title/Abstract] OR “health record*”[Title/Abstract] OR “healthcare record*”[Title/Abstract] OR “lab result*”[Title/Abstract] OR “laboratory result*”[Title/Abstract] OR “medical record*”[Title/Abstract] OR “mental health notes”[Title/Abstract] OR “patient record*”[Title/Abstract] OR “psychiatric notes”[Title/Abstract] OR “psychotherapy notes”[Title/Abstract] OR “registry”[Title/Abstract] OR “registries”[Title/Abstract] OR “visit notes”[Title/Abstract]	525,750	
6	#4 OR #5	689,256	
7	“Artificial Intelligence”[Mesh]	187,271	
8	“algorithm*”[Title/Abstract] OR “artificial intelligence”[Title/Abstract] OR “automat*”[Title/Abstract] OR “computational intelligence”[Title/Abstract] OR “data mining”[Title/Abstract] OR “deep learning”[Title/Abstract] OR “deep phenotyp*”[Title/Abstract] OR “digital phenotyp*”[Title/Abstract] OR “inferential statistic*”[Title/Abstract] OR “machine intelligence”[Title/Abstract] OR “machine learning”[Title/Abstract] OR “natural language process*”[Title/Abstract] OR “large language model*”[Title/Abstract] OR “neural network*”[Title/Abstract] OR “statistical inference*”[Title/Abstract]	810,156	
9	#7 OR #8	870,962	
10	“Attitude”[Mesh] OR “Patient Preference”[Mesh] OR “Physician-Patient Relations”[Mesh]	691,389	
11	acceptance[Title/Abstract] OR attitude*[Title/Abstract] OR barrier*[Title/Abstract] OR concerns[Title/Abstract] OR emotion*[Title/Abstract] OR expectation*[Title/Abstract] OR experience*[Title/Abstract] OR facilitat*[Title/Abstract] OR feedback[Title/Abstract] OR feeling*[Title/Abstract] OR interview*[Title/Abstract] OR opinion*[Title/Abstract] OR perception*[Title/Abstract] OR perspective*[Title/Abstract] OR “point of view*”[Title/Abstract] OR preference*[Title/Abstract]	4,227,463	
12	#10 OR #11	4,574,230	
13	#3 AND #6 AND #9 AND #12	2865	
**Scopus, 22 January 2024**			
**Search no**	**Search terms**	**Results**	**Comments**
1	TITLE-ABS-KEY (“classification of disease*” OR “diagnosis code*” OR phecode* OR phenotyp* OR (icd W/3 code OR codes OR coding) OR (dsm W/3 code OR codes OR coding))	1,110,187	
2	TITLE-ABS-KEY (“clinical data” OR “clinic notes” OR “clinical notes” OR “progress notes” OR “diagnosis code*” OR “doctors notes” OR “EHR” OR “EMR” OR “health data” OR “health electronic record*” OR “health record*” OR “healthcare record*” OR “lab result*” OR “laboratory result*” OR “medical record*” OR “mental health notes” OR “patient record*” OR “psychiatric notes” OR “psychotherapy notes” OR “registry” OR “registries” OR “visit notes”)	900,545	
3	TITLE-ABS-KEY (“algorithm*” OR “artificial intelligence” OR “automat*” OR “computational intelligence” OR “data mining” OR “deep learning” OR “deep phenotyp*” OR “digital phenotyp*” OR “inferential statistic*” OR “machine intelligence” OR “machine learning” OR “natural language process*” OR “large language model*” OR “neural network*” OR “statistical inference*”)	6,679,667	
4	TITLE-ABS-KEY (acceptance OR attitude* OR barrier* OR concerns OR emotion* OR expectation* OR experience* OR facilitat* OR feedback OR feeling* OR interview* OR opinion* OR perception* OR perspective* OR “point of view*” OR preference*)	12,352,535	
5	#1 AND #2 AND #3 AND #4	916	
**Web of Science Core Collection, 22 January 2024**			
**Search no**	**Search terms**	**Results**	**Comments**
1	TS = (“classification of disease*” OR “diagnosis code*” OR phecode* OR phenotyp* OR (icd NEAR/3 (code OR codes OR coding)) OR (dsm NEAR/3 (code OR codes OR coding)))	867,030	
2	TS = (“clinical data” OR “clinic notes” OR “clinical notes” OR “progress notes” OR “diagnosis code*” OR “doctors notes” OR “EHR” OR “EMR” OR “health data” OR “health electronic record*” OR “health record*” OR “healthcare record*” OR “lab result*” OR “laboratory result*” OR “medical record*” OR “mental health notes” OR “patient record*” OR “psychiatric notes” OR “psychotherapy notes” OR “registry” OR “registries” OR “visit notes”)	529,945	
3	TS = (“algorithm*” OR “artificial intelligence” OR “automat*” OR “computational intelligence” OR “data mining” OR “deep learning” OR “deep phenotyp*” OR “digital phenotyp*” OR “inferential statistic*” OR “machine intelligence” OR “machine learning” OR “natural language process*” OR “large language model*” OR “neural network*” OR “statistical inference*”)	4,255,897	
4	TS = (acceptance OR attitude* OR barrier* OR concerns OR emotion* OR expectation* OR experience* OR facilitat* OR feedback OR feeling* OR interview* OR opinion* OR perception* OR perspective* OR “point of view*” OR preference*)	9,237,009	
5	#1 AND #2 AND #3 AND #4	624	

(*) is a truncation (wildcard) symbol. It tells the database to search for all words that begin with the specified root and have any ending.
